# Stacking order as a tuning parameter in layered quantum materials

**DOI:** 10.1093/nsr/nwag560

**Published:** 2026-09-07

**Authors:** Linpeng Nie, Tao Wu

**Affiliations:** Hefei National Research Center for Physical Sciences at the Microscale, University of Science and Technology of China, China; Hefei National Research Center for Physical Sciences at the Microscale, University of Science and Technology of China, China; CAS Key Laboratory of Strongly coupled Quantum Matter Physics, Department of Physics, University of Science and Technology of China, China; Collaborative Innovation Center of Advanced Microstructures, Nanjing University, China; Hefei National Laboratory, University of Science and Technology of China, China

In layered quantum materials, chemical composition is only part of the story. Equally important is how identical atomic layers are arranged with respect to one another. Collectively referred to as stacking order, these structural relationships include the lateral registry between neighboring layers and the sequence in which different layers are stacked. Increasing evidence shows that stacking order can profoundly reshape electronic structures and quantum phases without altering chemical composition [1–3].

Recent progress has expanded stacking engineering from registry control to sequence control. Twisting and interlayer sliding manipulate the relative positions of neighboring layers in two-dimensional van der Waals materials [4], whereas artificial superstructures engineer the stacking sequence of different structural building blocks of oxides [5]. Together, these advances have transformed stacking order from a static crystallographic descriptor into an active tuning parameter for quantum-state engineering, offering new possibilities for dynamically reconfigurable and ultimately programmable quantum materials.

Yet both stacking registry and stacking sequence are generally predetermined during crystal growth or artificial assembly. Consequently, stacking engineering has remained largely a static approach, lacking the capability for structural evolution within a chemically uniform crystal. Adaptive stacking in endotaxial heterostructures represents a route beyond static stacking engineering by allowing stacking order to evolve spontaneously within a chemically uniform crystal, while simultaneously creating heterogeneous interfaces with competing electronic orders.

Cao and co-workers now demonstrate this concept in bulk TaS_2_ by realizing an adaptive stacking pathway [6]. Unlike externally imposed twisting or artificial assembly, adaptive stacking originates from spontaneous structural evolution within the crystal itself. Their previous work showed that collective interlayer sliding alone can switch 1T-TaS_2_ between a three-dimensional band insulator and a two-dimensional Mott insulator by tuning interlayer hybridization [7]. Building on this discovery, the present study demonstrates that stacking order itself can spontaneously evolve through coordinated structural reconstruction. Thermal annealing triggers a sequence of layer-resolved structural transformations, converting selected 1T layers into 1H or 1H′ phases while preserving the overall crystal framework. Collective interlayer sliding first reconstructs the stacking registry within the 1T phase, after which layer-specific atomic rearrangements generate ordered hetero-phase superlattices, including paired 1H/1T and sandwiched 1H/1T/1H′ configurations (Fig. 1a). The stacking sequence is therefore generated *in situ*, rather than predetermined solely by crystal growth or artificial assembly.

**Figure 1.**
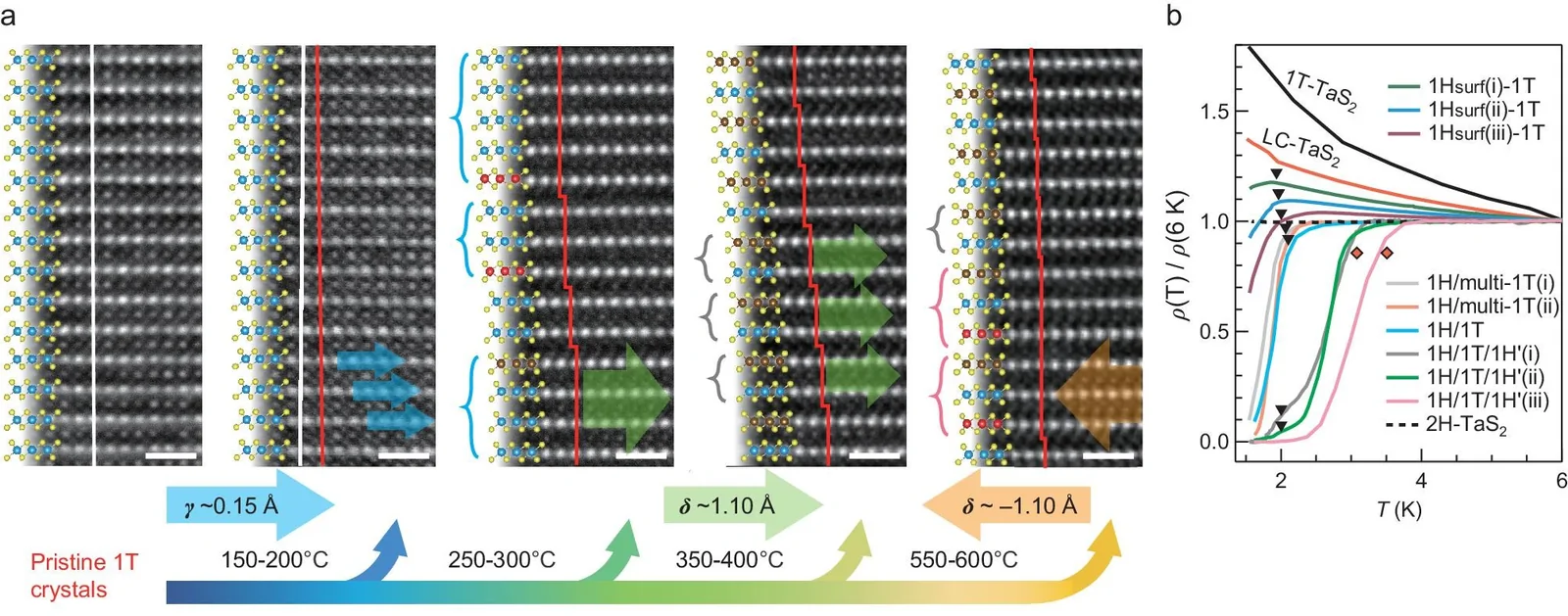
Adaptive stacking and emergent quantum phases in bulk TaS_2_. (a) Aberration-corrected high-angle annular dark-field scanning transmission electron microscopy images illustrating the thermally driven *in situ* evolution of stacking configurations and the formation of hetero-phase superlattices. (b) Temperature-dependent in-plane resistivity demonstrating stacking-dependent superconducting transitions. Adapted with permission from Ref. [6].

The authors attribute this adaptive evolution to a two-stage sliding mechanism. Low-barrier collective interlayer sliding first reconstructs the stacking registry and modifies the local interlayer environment. Subsequent displacement of a sulfur atomic plane changes the local Ta coordination from 1T to 1H. Repetition of these processes, together with structural relaxation, produces the observed hetero-phase superlattices.

More importantly, the emergent stacking sequences are not merely structural variants but create distinct electronic environments. Inter-phase charge transfer progressively suppresses the charge-density-wave state in the 1T layers, with the sandwiched 1H/1T/1H′ configuration producing stronger charge redistribution than the paired 1H/1T structure. Correspondingly, the system undergoes an insulating-to-superconducting electronic phase evolution (Fig. 1b), while the paired 1H/1T and sandwiched 1H/1T/1H′ superlattices exhibit distinct superconducting transitions near 2 and 3 K, respectively. This sequence-dependent evolution of charge order and superconductivity establishes stacking sequence itself as an effective tuning parameter for correlated quantum phases.

The authors propose a two-stage sliding mechanism in which low-barrier collective interlayer sliding first reconstructs the stacking registry, followed by local sulfur-plane displacement that converts the Ta coordination from 1T to 1H, generating the observed hetero-phase superlattices.

More broadly, adaptive stacking introduces a new materials design principle in which structural evolution itself, rather than chemical composition, becomes an active variable for engineering correlated quantum phases without changing the chemical composition. An important next step will be to determine whether such stacking sequences can be written locally and switched reversibly. Achieving this level of control would transform adaptive stacking into genuinely programmable stacking, opening a route toward reconfigurable quantum materials in which electronic phases can be selected and reconfigured through atomic-scale structural sequences.
